# Supplementary figures and images for: Alternative splicing of apoptosis genes promotes human T cell survival
Source: eLife. 2022 Oct 20;11:e80953. doi: 10.7554/eLife.80953 (PMC9625086; doi:10.7554/eLife.80953)

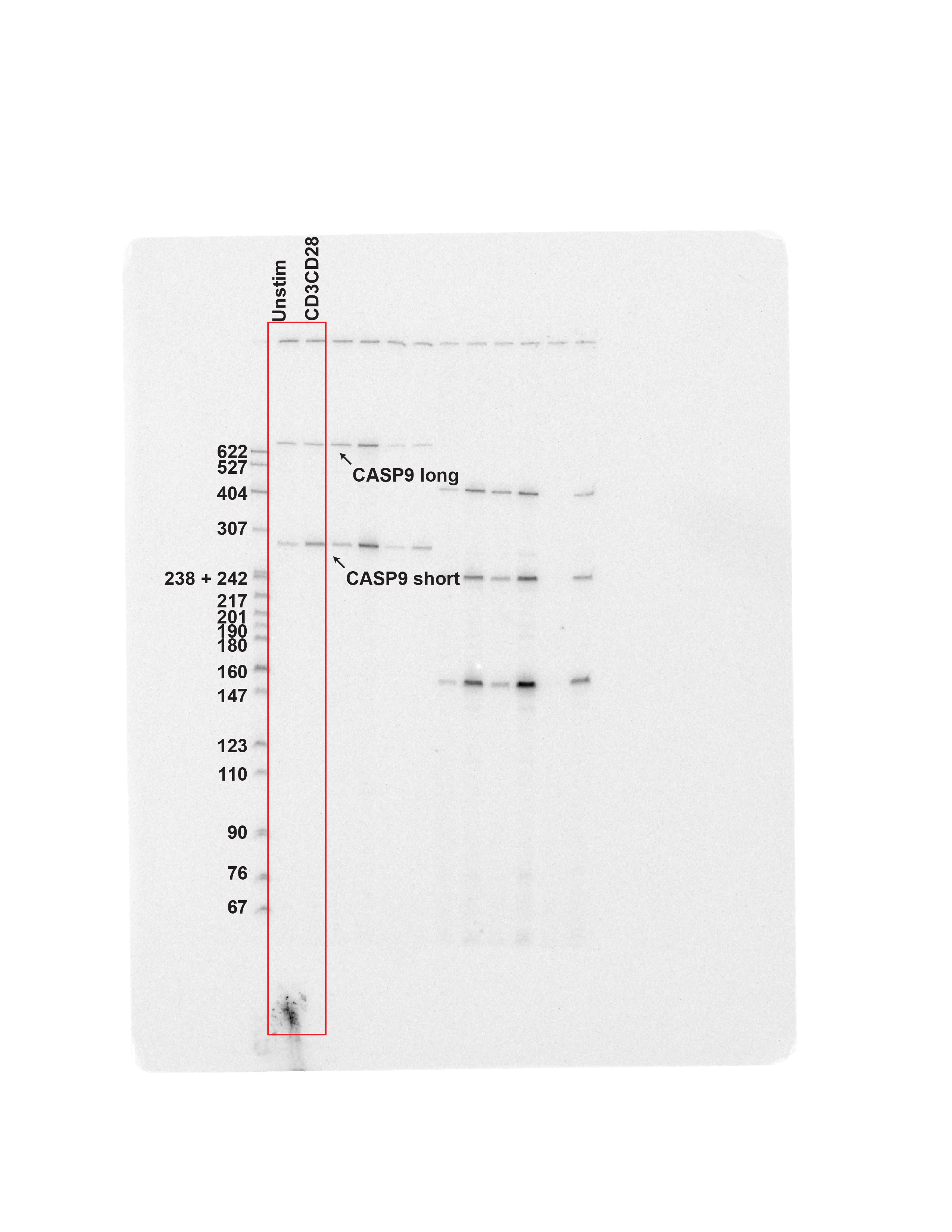

Supplement: Figure 4—source data 1. [file elife-80953-fig4-data1.zip › Figure 4 - source data 1 - CASP9 labeled - Figure 4B.jpg]

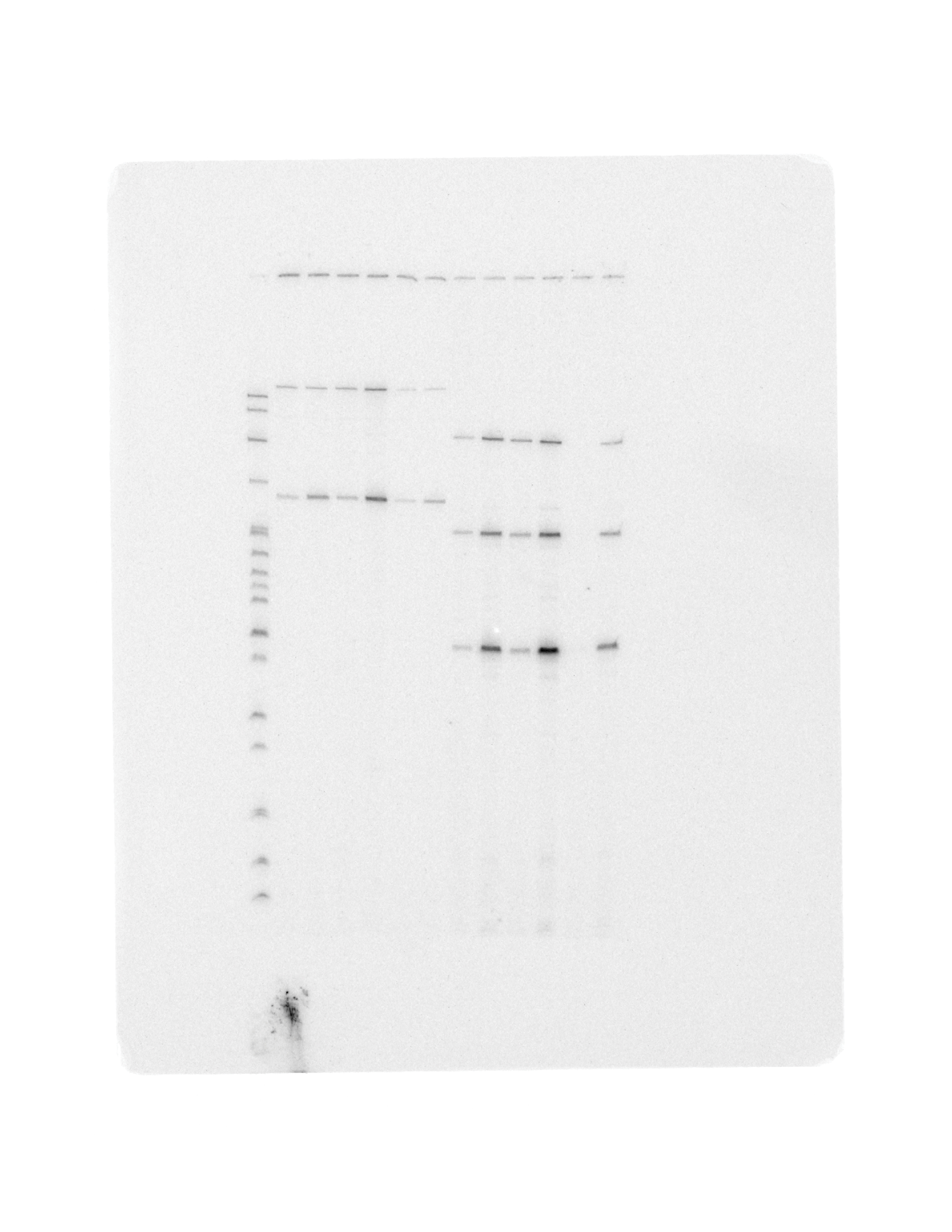

Supplement: Figure 4—source data 1. [file elife-80953-fig4-data1.zip › Figure 4 - source data 1 - CASP9 unlabeled - Figure 4B.jpg]

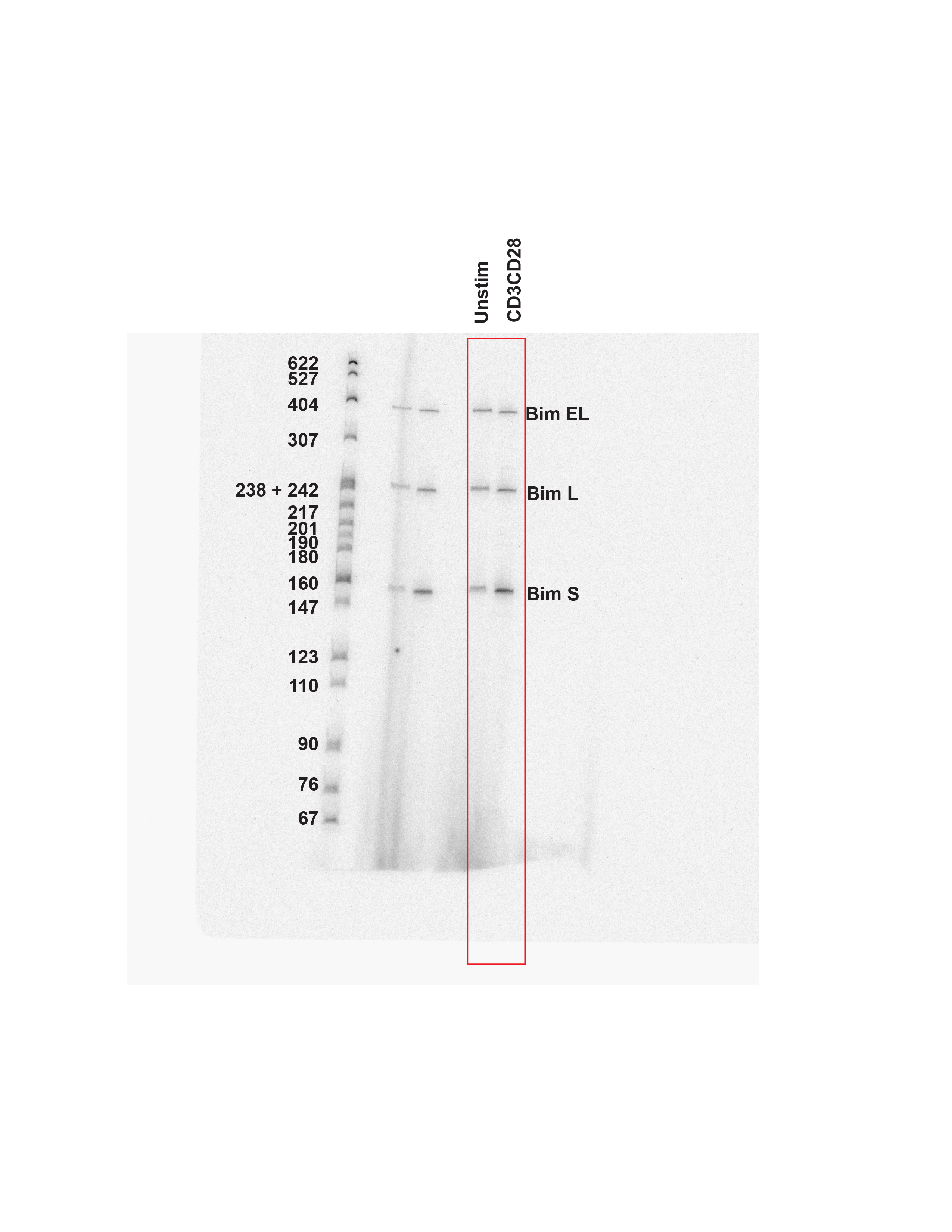

Supplement: Figure 4—source data 2. [file elife-80953-fig4-data2.zip › Figure 4 - source data 2 - Bim labeled - Figure 4C.jpg]

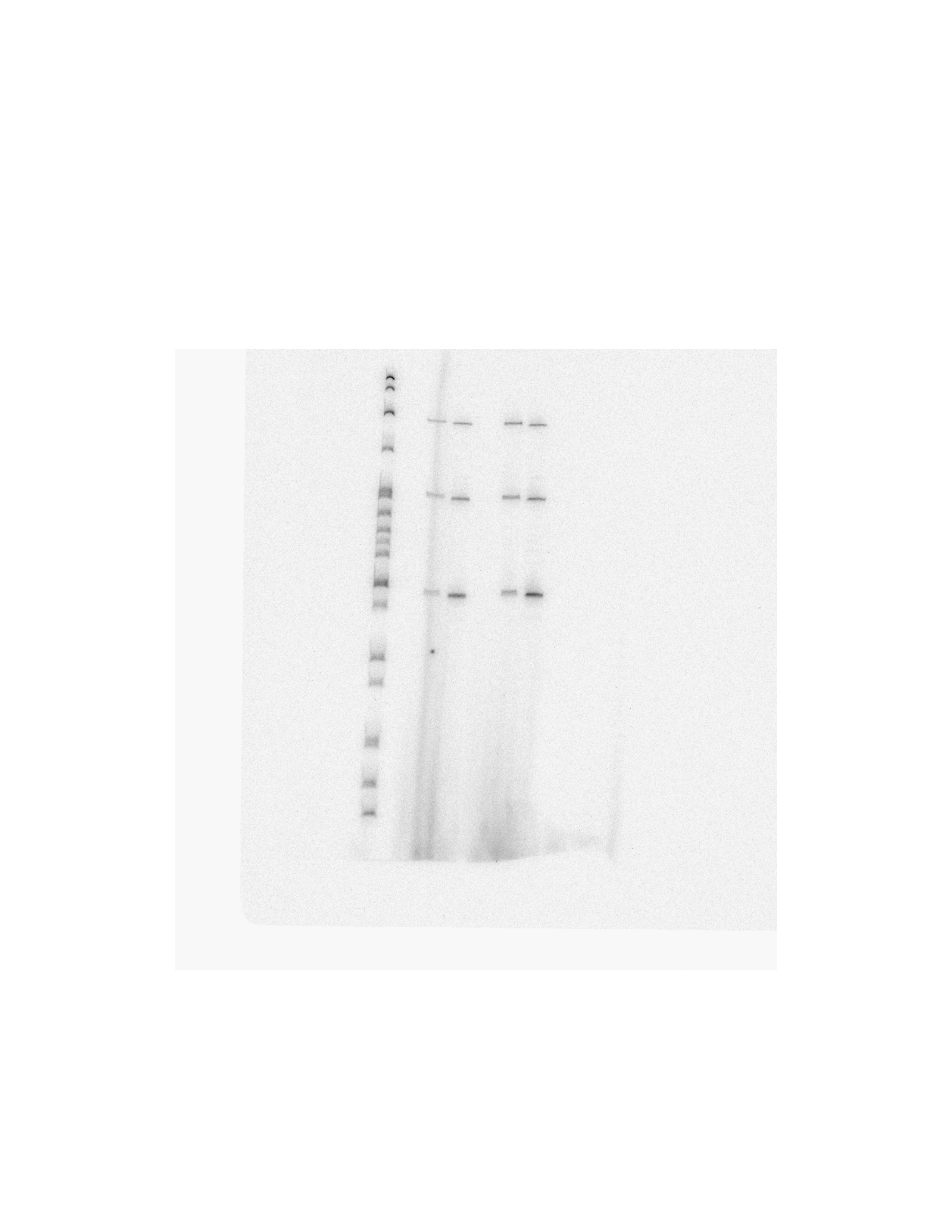

Supplement: Figure 4—source data 2. [file elife-80953-fig4-data2.zip › Figure 4 - source data 2 - Bim unlabeled - Figure 4C.jpg]

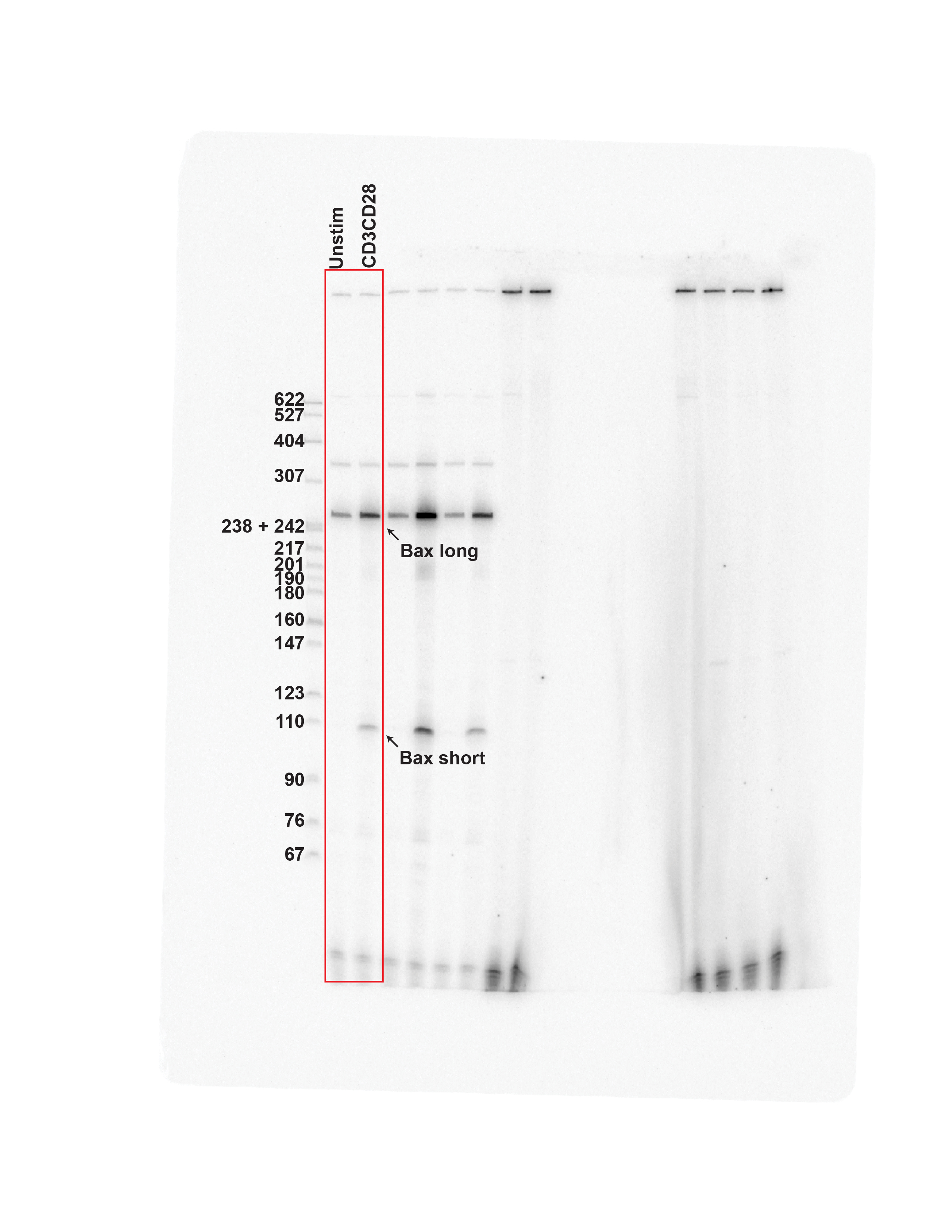

Supplement: Figure 4—source data 3. [file elife-80953-fig4-data3.zip › Figure 4 - source data 3 - Bax labeled - Figure 4D.jpg]

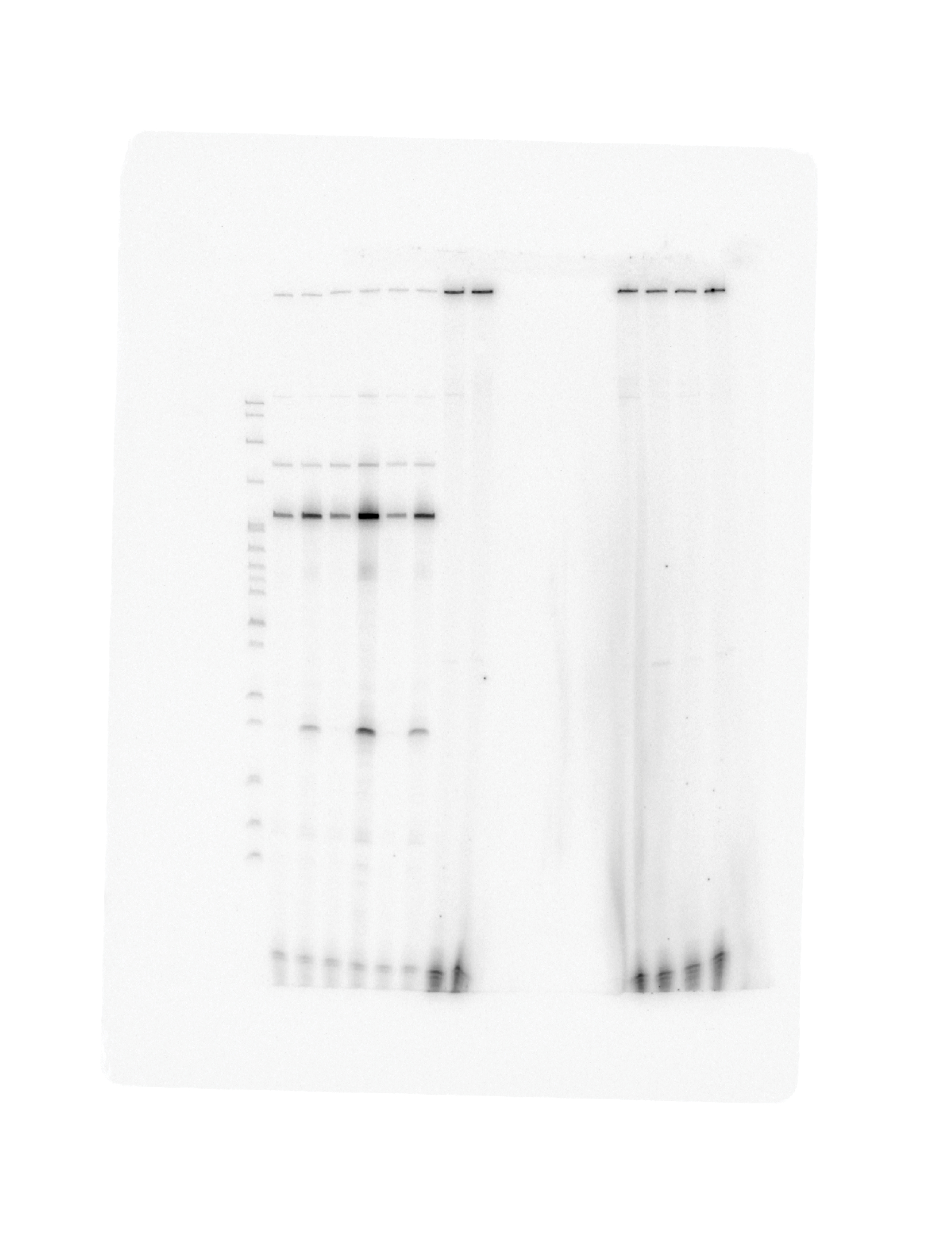

Supplement: Figure 4—source data 3. [file elife-80953-fig4-data3.zip › Figure 4 - source data 3 - Bax unlabeled - Figure 4D.jpg]

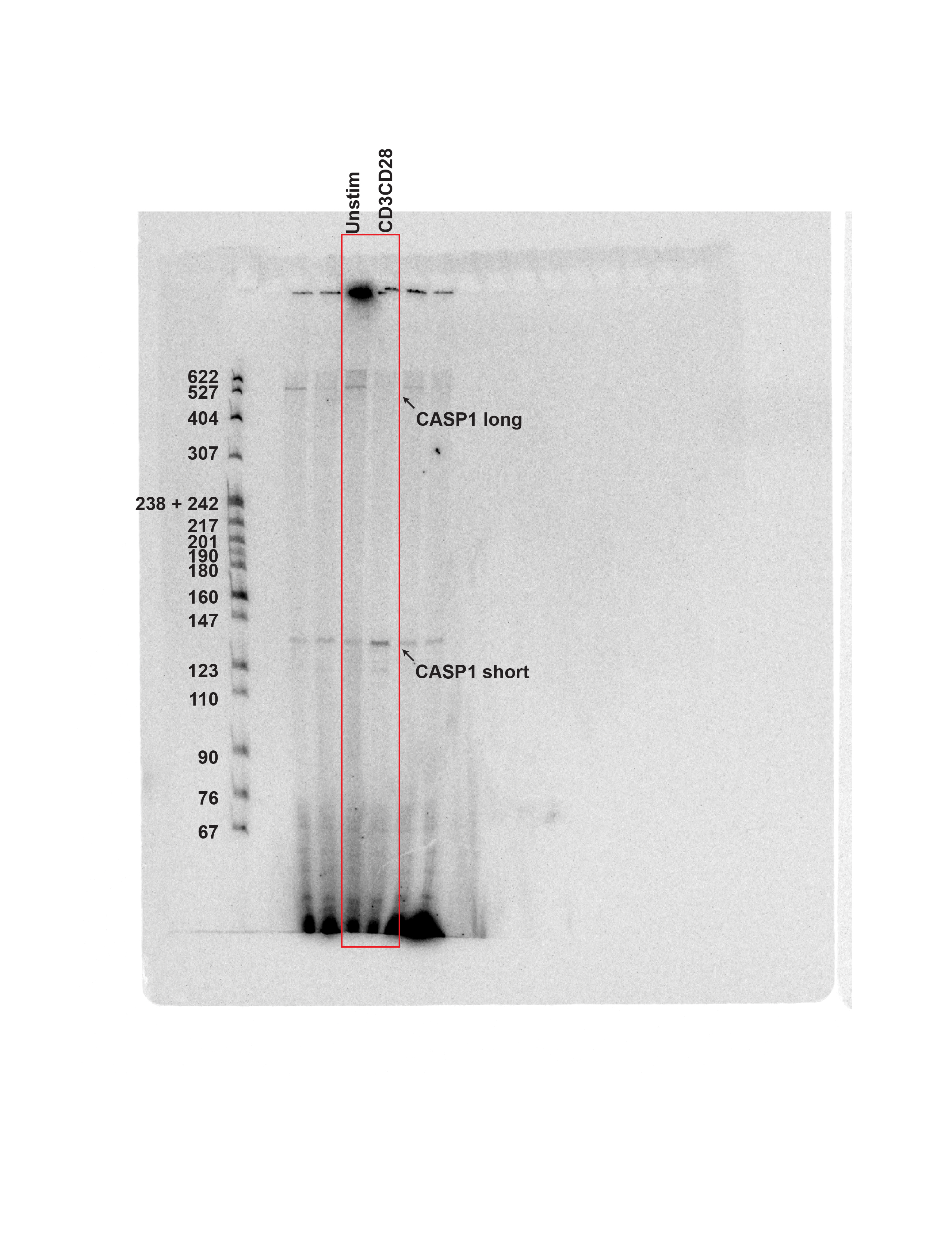

Supplement: Figure 4—source data 4. [file elife-80953-fig4-data4.zip › Figure 4 - source data 4 - CASP1 labeled - Figure 4E.jpg]

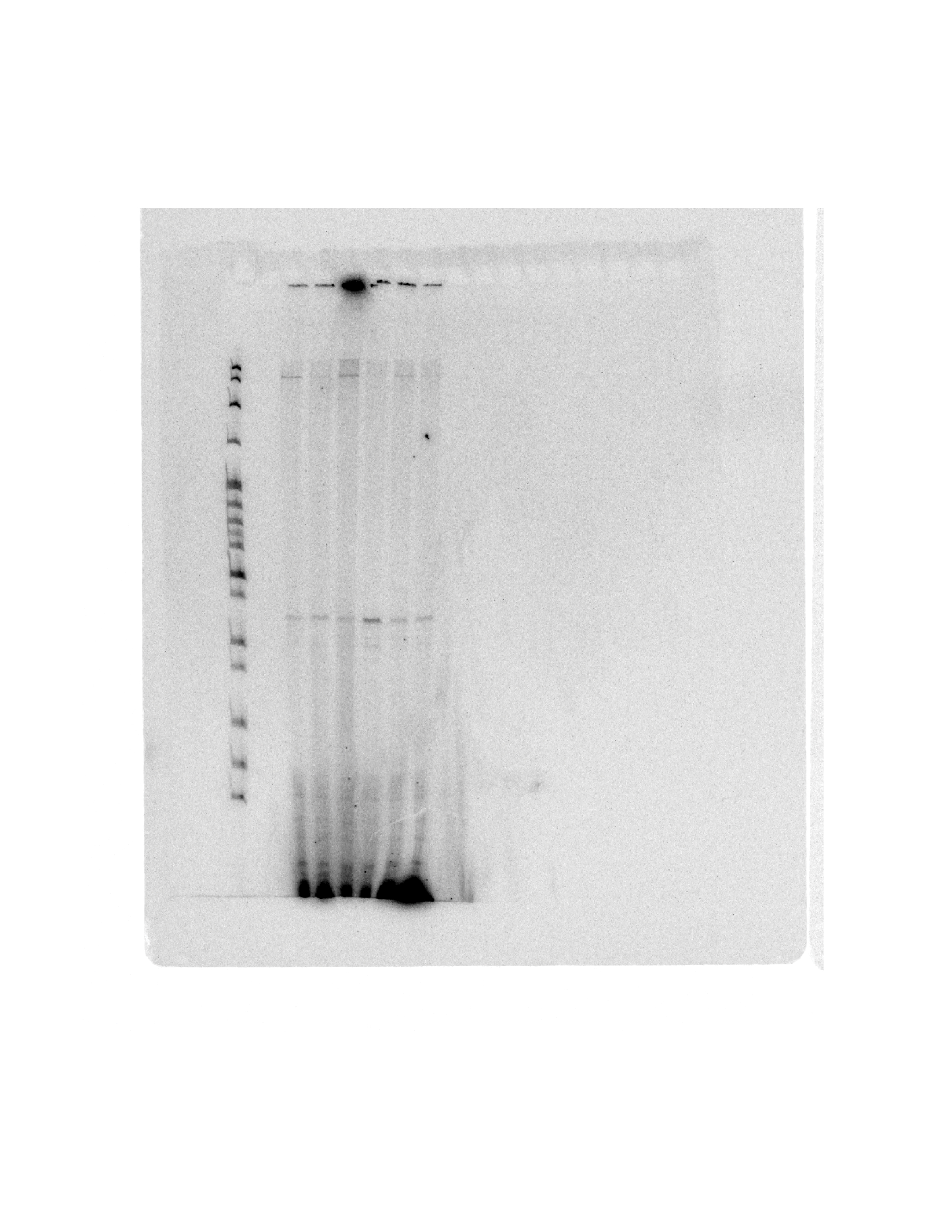

Supplement: Figure 4—source data 4. [file elife-80953-fig4-data4.zip › Figure 4 - source data 4 - CASP1 unlabeled - Figure 4E.jpg]

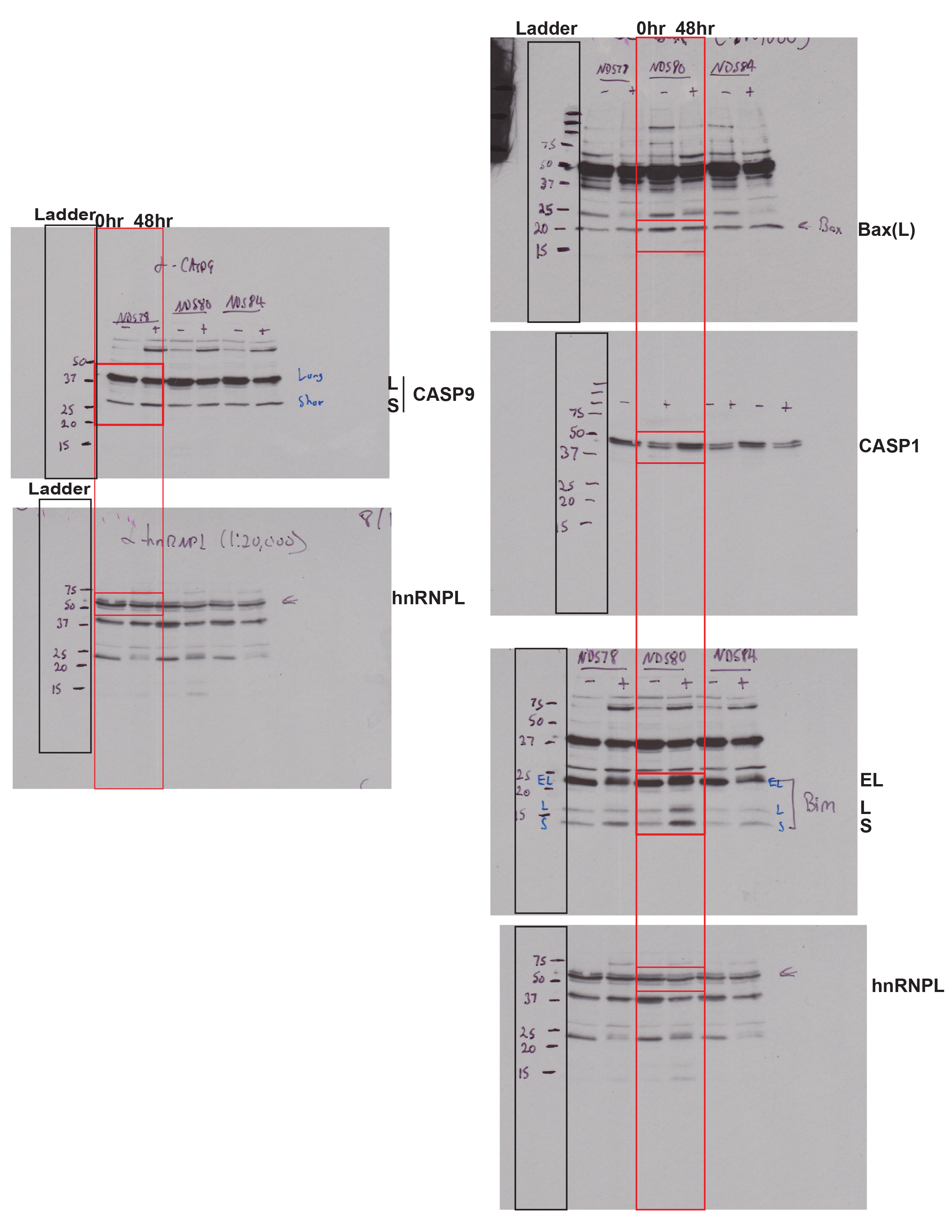

Supplement: Figure 4—source data 5. [file elife-80953-fig4-data5.zip › Figure 4 - source data 5 - westerns labeled - Figure 4F.jpg]

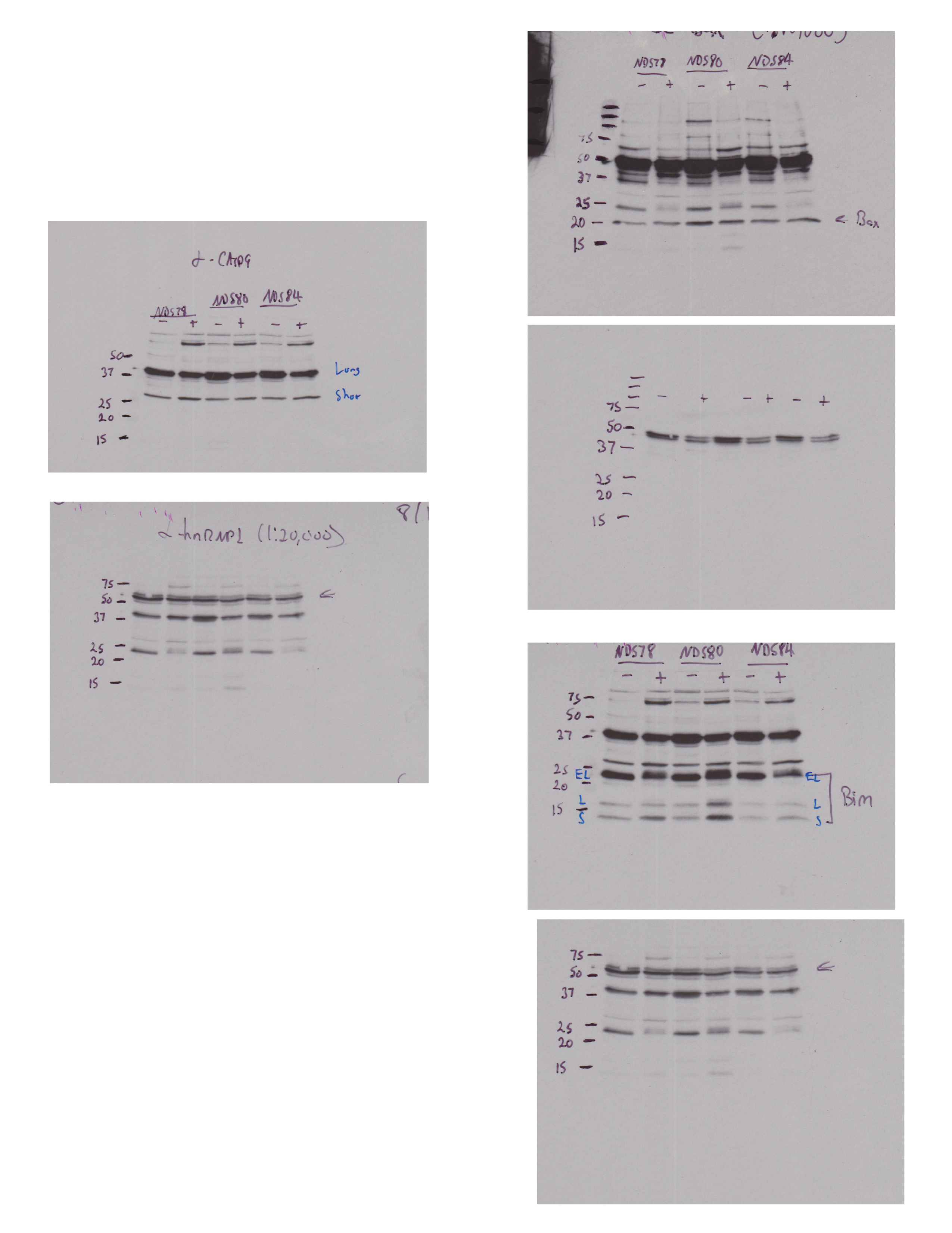

Supplement: Figure 4—source data 5. [file elife-80953-fig4-data5.zip › Figure 4 - source data 5 - westerns unlabeled - Figure 4F.jpg]

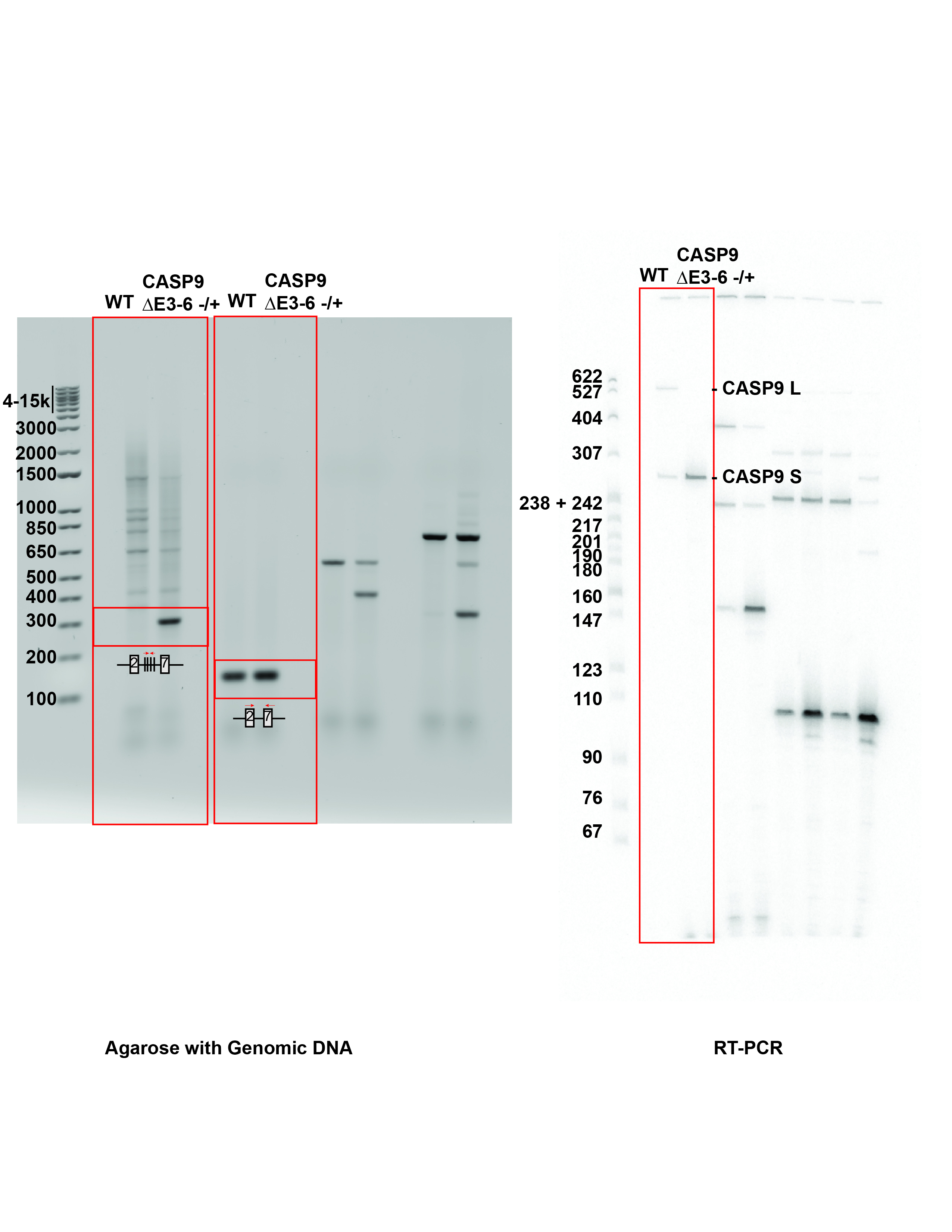

Supplement: Figure 5—source data 1. [file elife-80953-fig5-data1.zip › Figure 5 - source data 1 - CASP9 labeled - 5A.jpg]

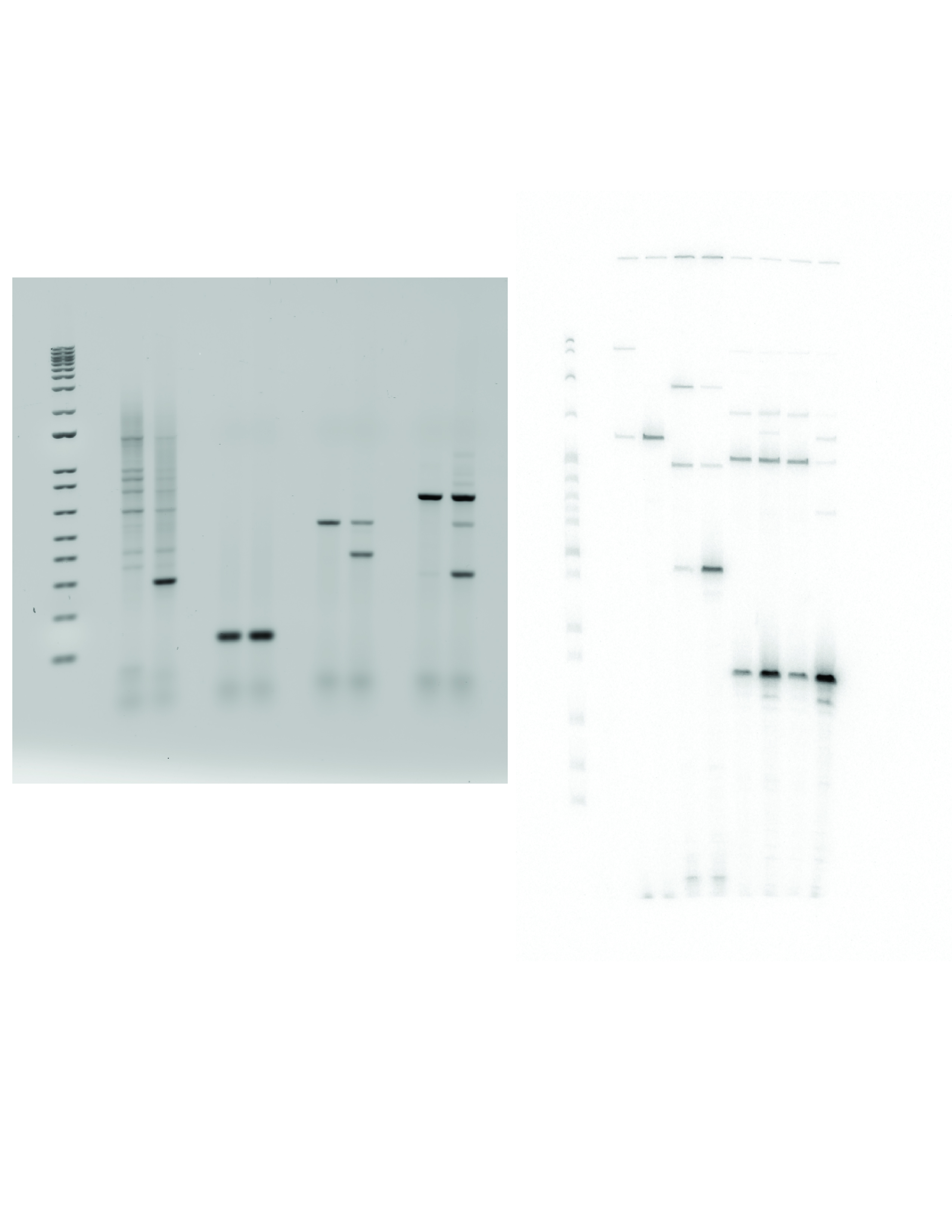

Supplement: Figure 5—source data 1. [file elife-80953-fig5-data1.zip › Figure 5 - source data 1 - CASP9 unlabeled - 5A.jpg]

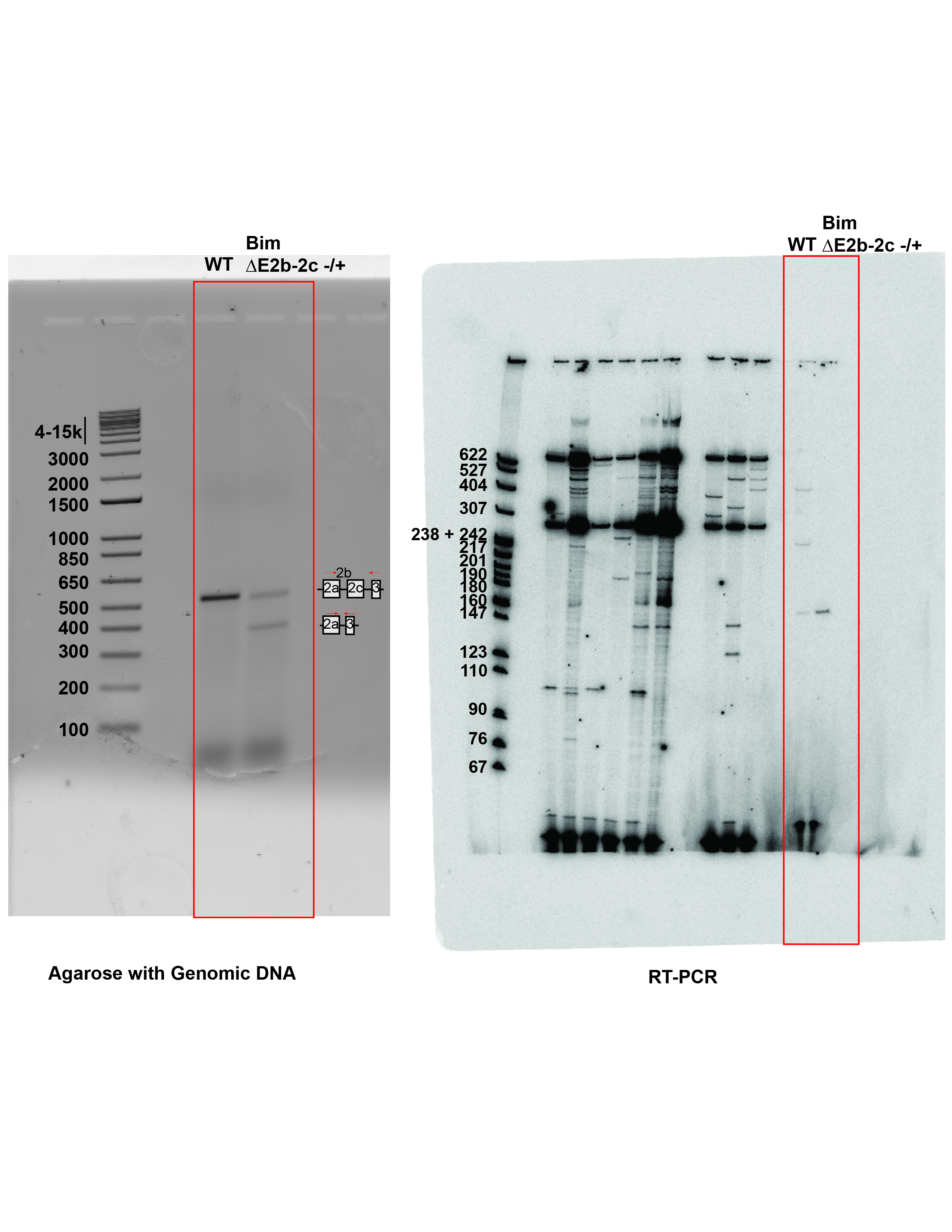

Supplement: Figure 5—source data 2. [file elife-80953-fig5-data2.zip › Figure 5 - source data 2 - Bim labeled - 5D.jpg]

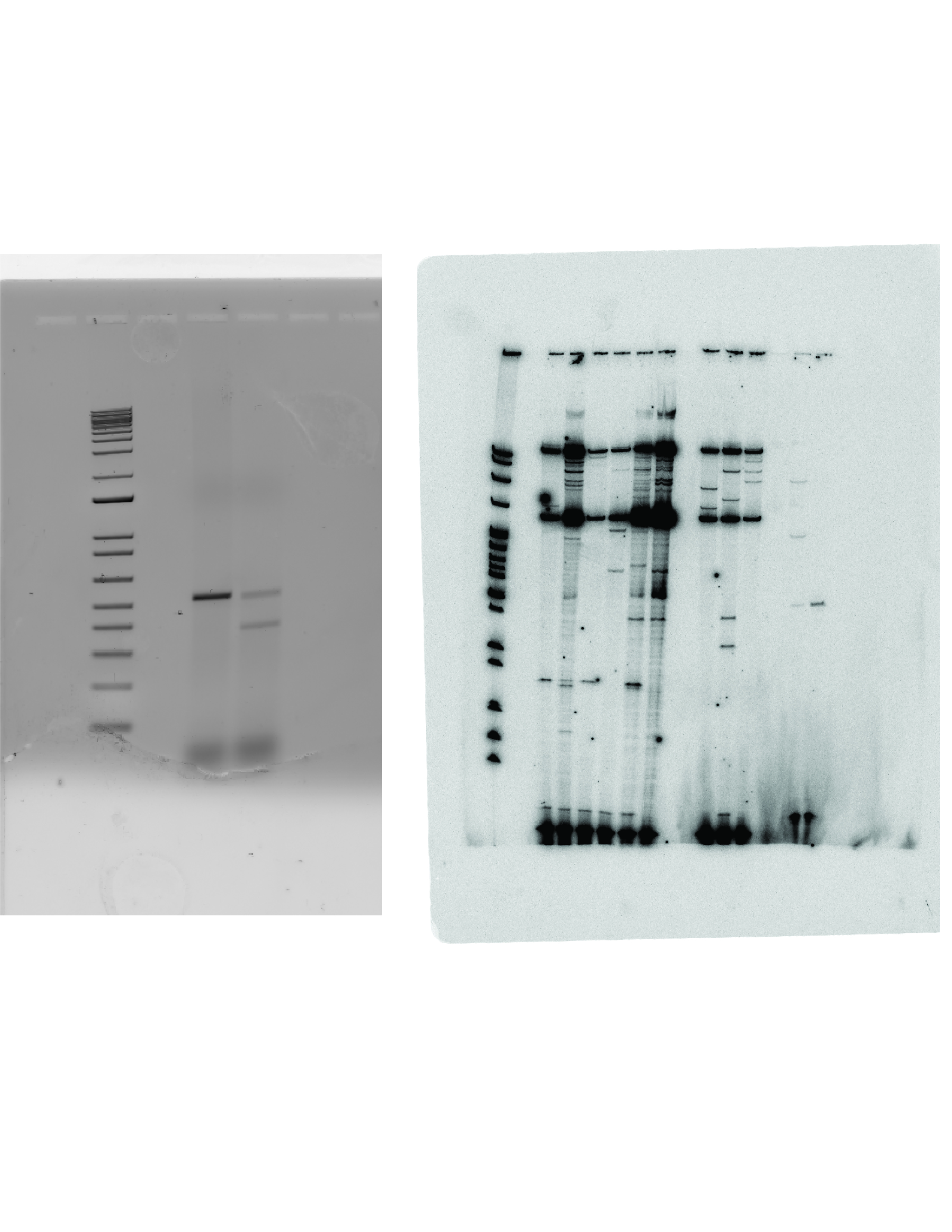

Supplement: Figure 5—source data 2. [file elife-80953-fig5-data2.zip › Figure 5 - source data 2 - Bim unlabeled - 5D.jpg]

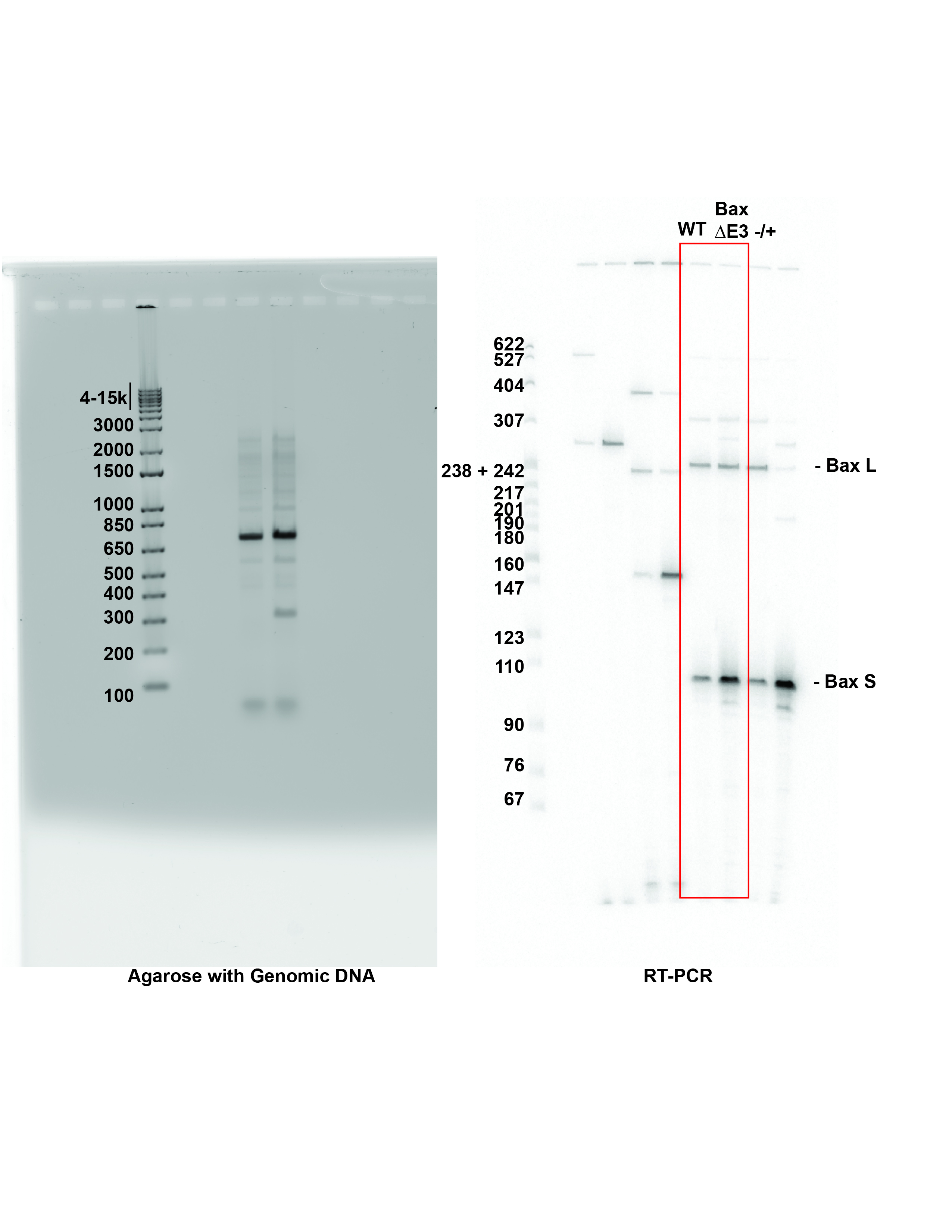

Supplement: Figure 5—source data 3. [file elife-80953-fig5-data3.zip › Figure 5 - source data 3 - Bax labeled - 5H.jpg]

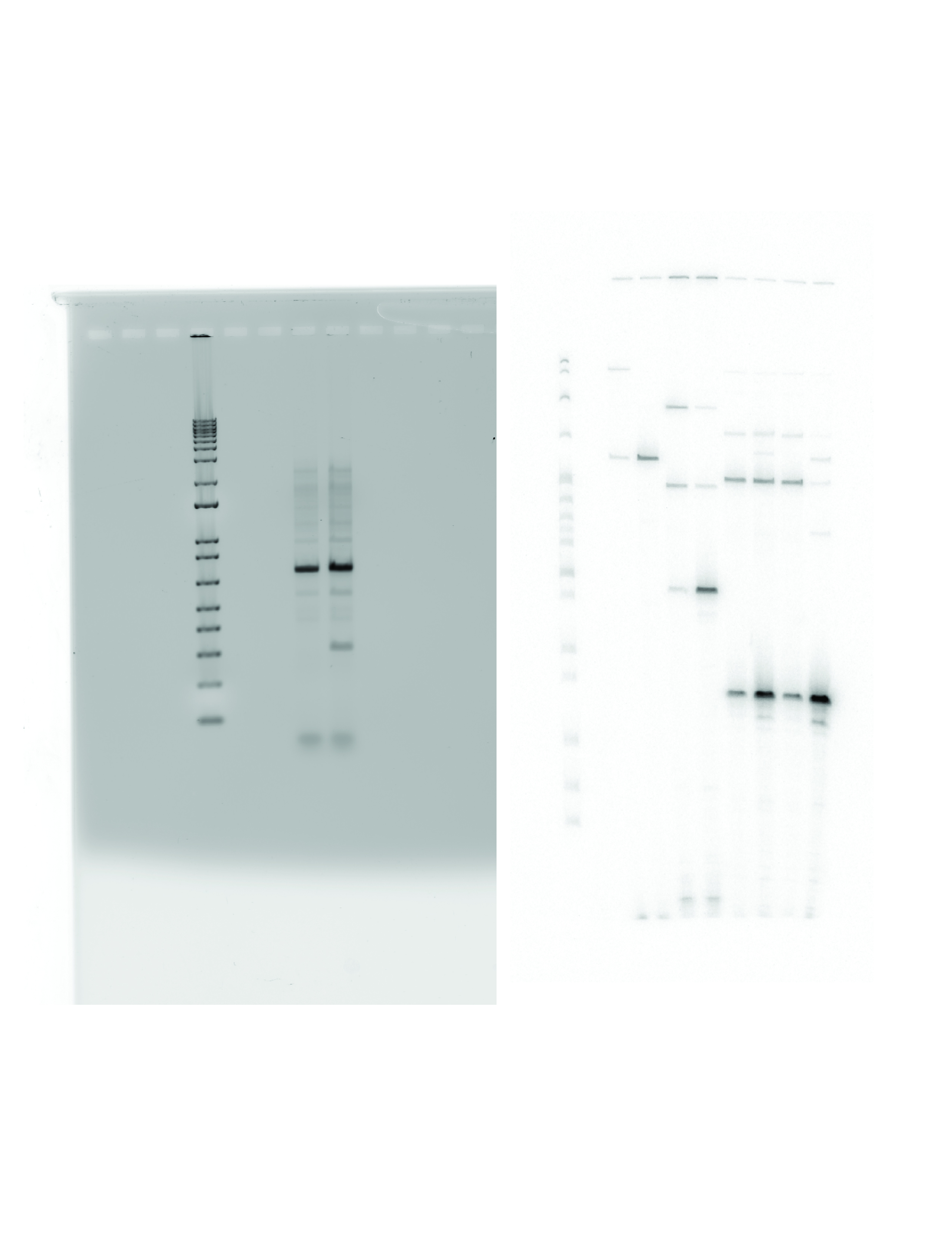

Supplement: Figure 5—source data 3. [file elife-80953-fig5-data3.zip › Figure 5 - source data 3 - Bax unlabeled - 5H.jpg]

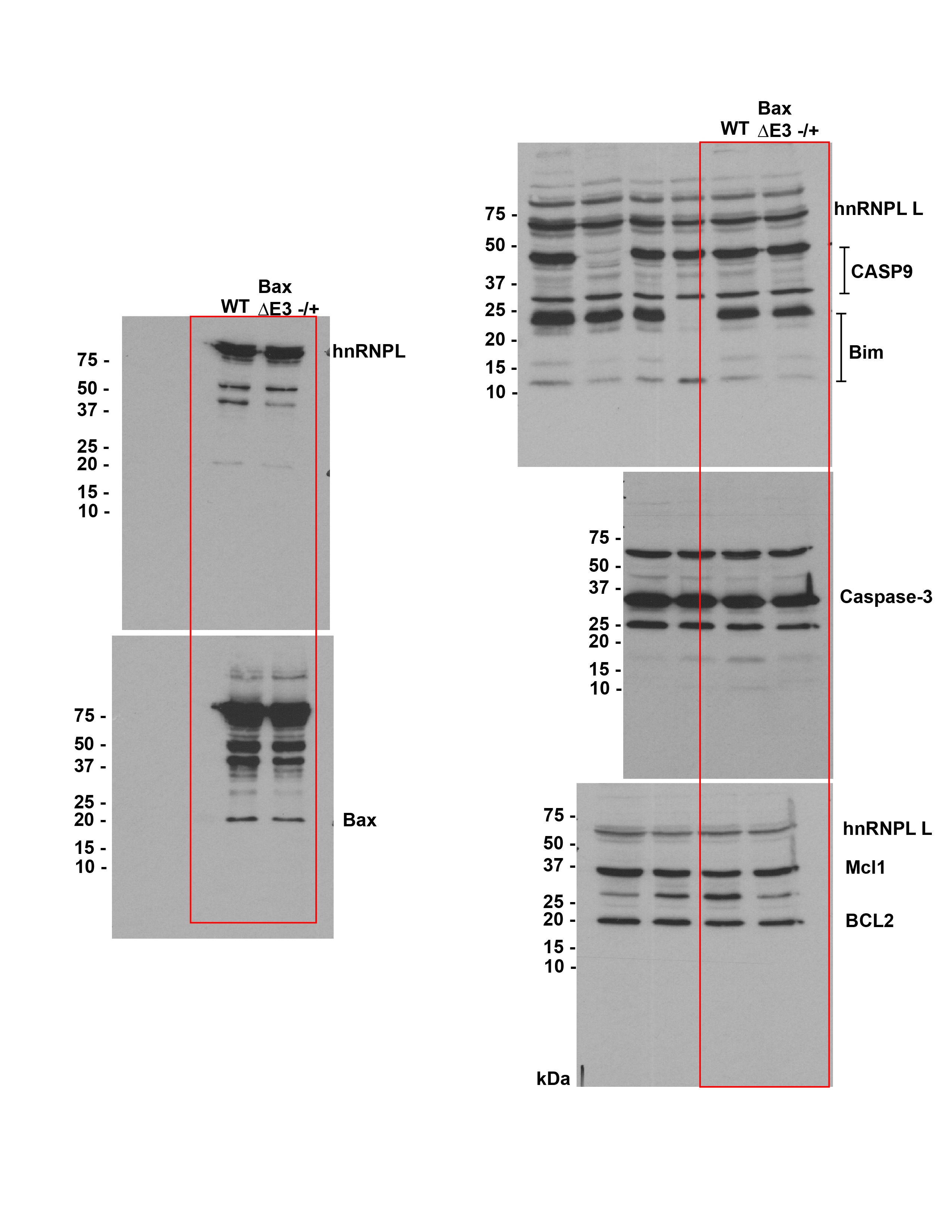

Supplement: Figure 5—figure supplement 1—source data 1. [file elife-80953-fig5-figsupp1-data1.zip › Figure 5 - figure supplemental 1 - source data 1 - Bax clone - labeled.jpg]

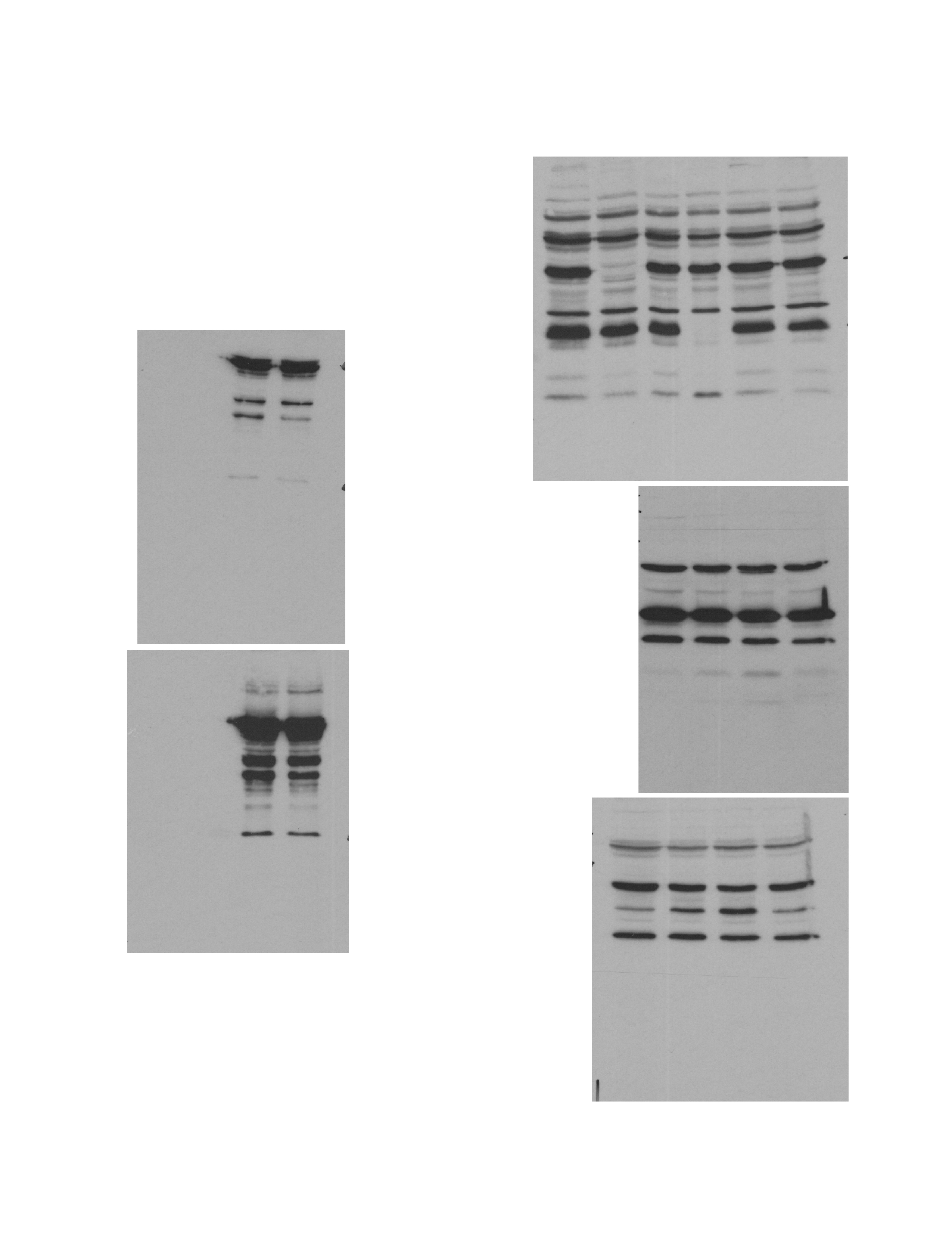

Supplement: Figure 5—figure supplement 1—source data 1. [file elife-80953-fig5-figsupp1-data1.zip › Figure 5 - figure supplemental 1 - source data 1 - Bax clone - unlabeled.jpg]

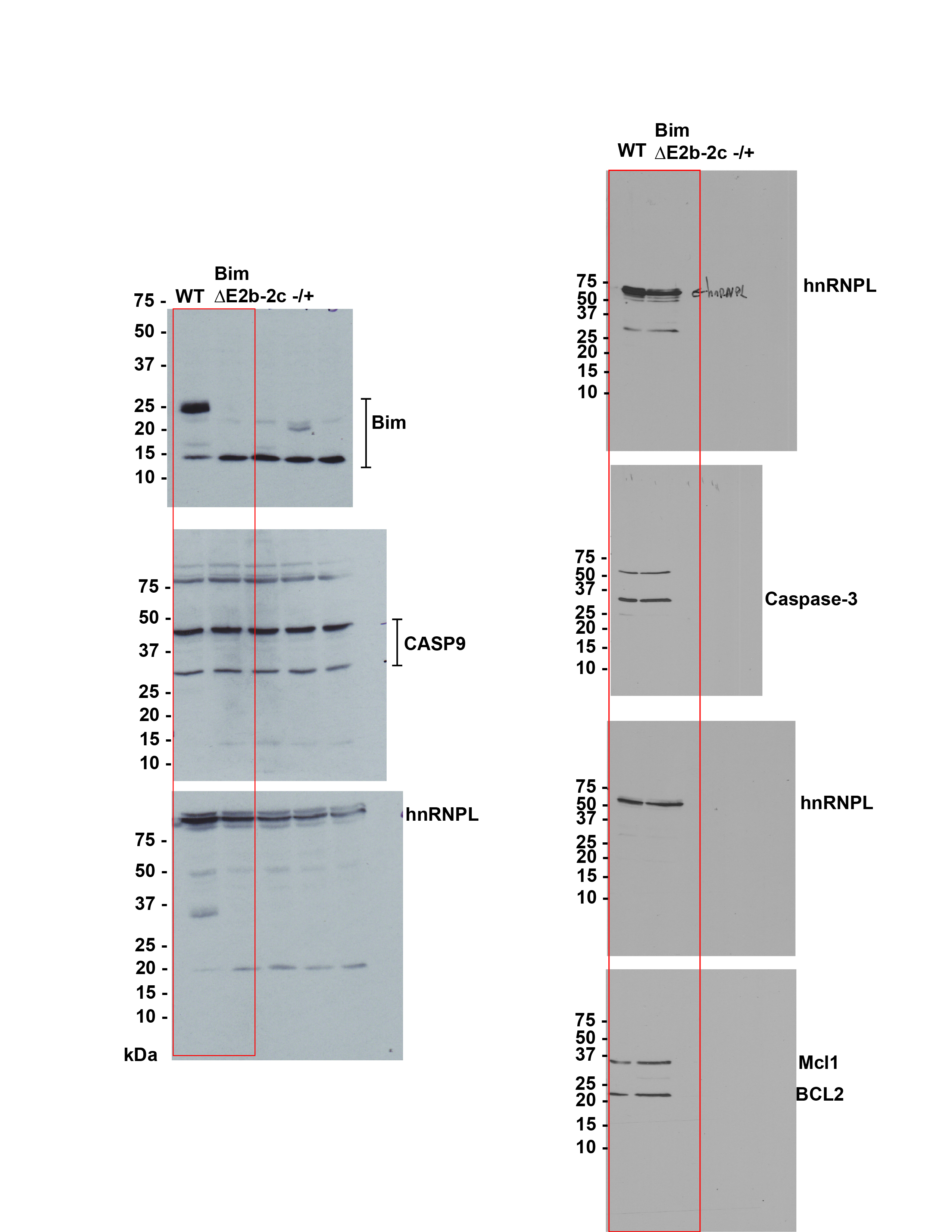

Supplement: Figure 5—figure supplement 1—source data 1. [file elife-80953-fig5-figsupp1-data1.zip › Figure 5 - figure supplemental 1 - source data 1 - Bim clone - labeled.jpg]

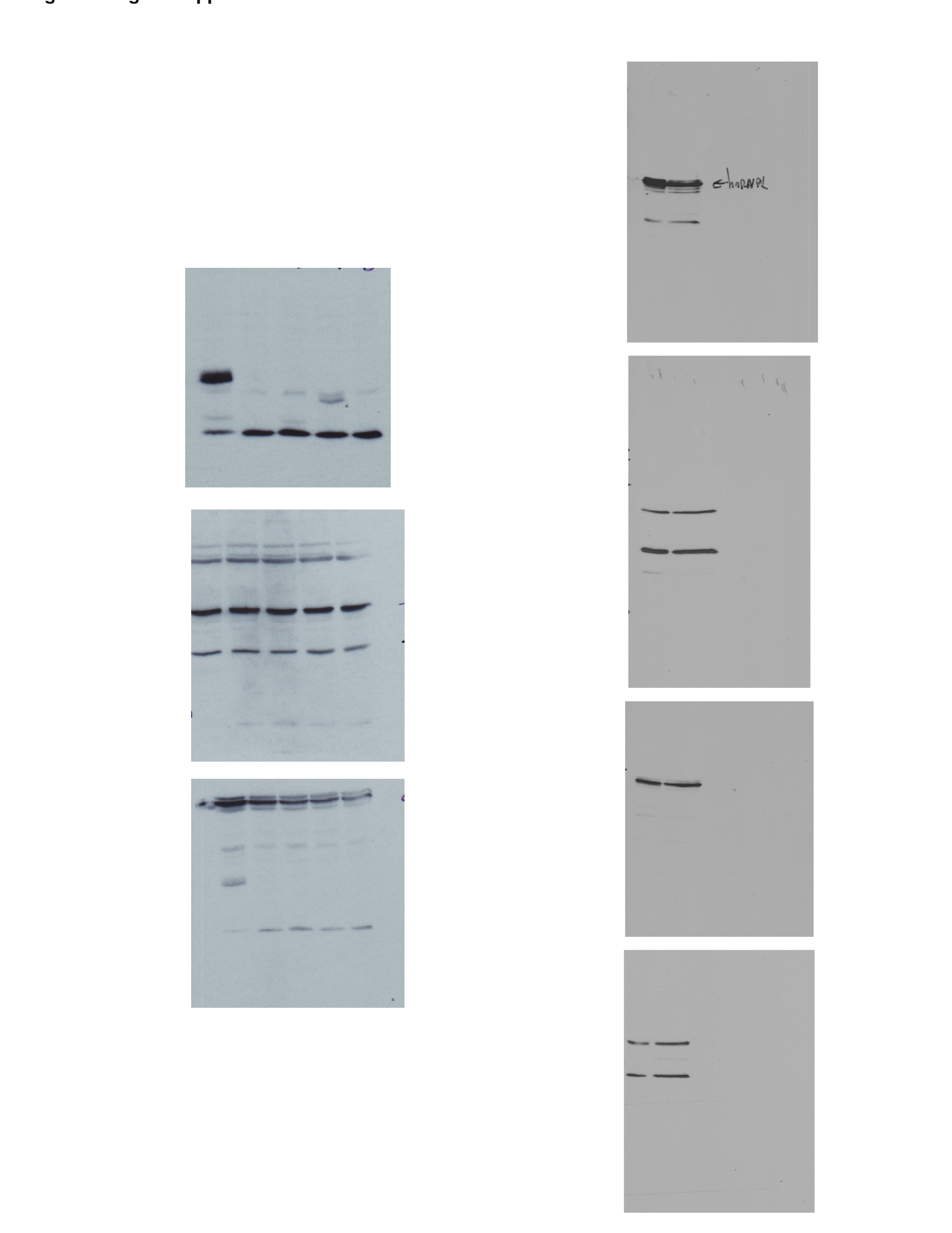

Supplement: Figure 5—figure supplement 1—source data 1. [file elife-80953-fig5-figsupp1-data1.zip › Figure 5 - figure supplemental 1 - source data 1 - Bim clone - unlabeled.jpg]

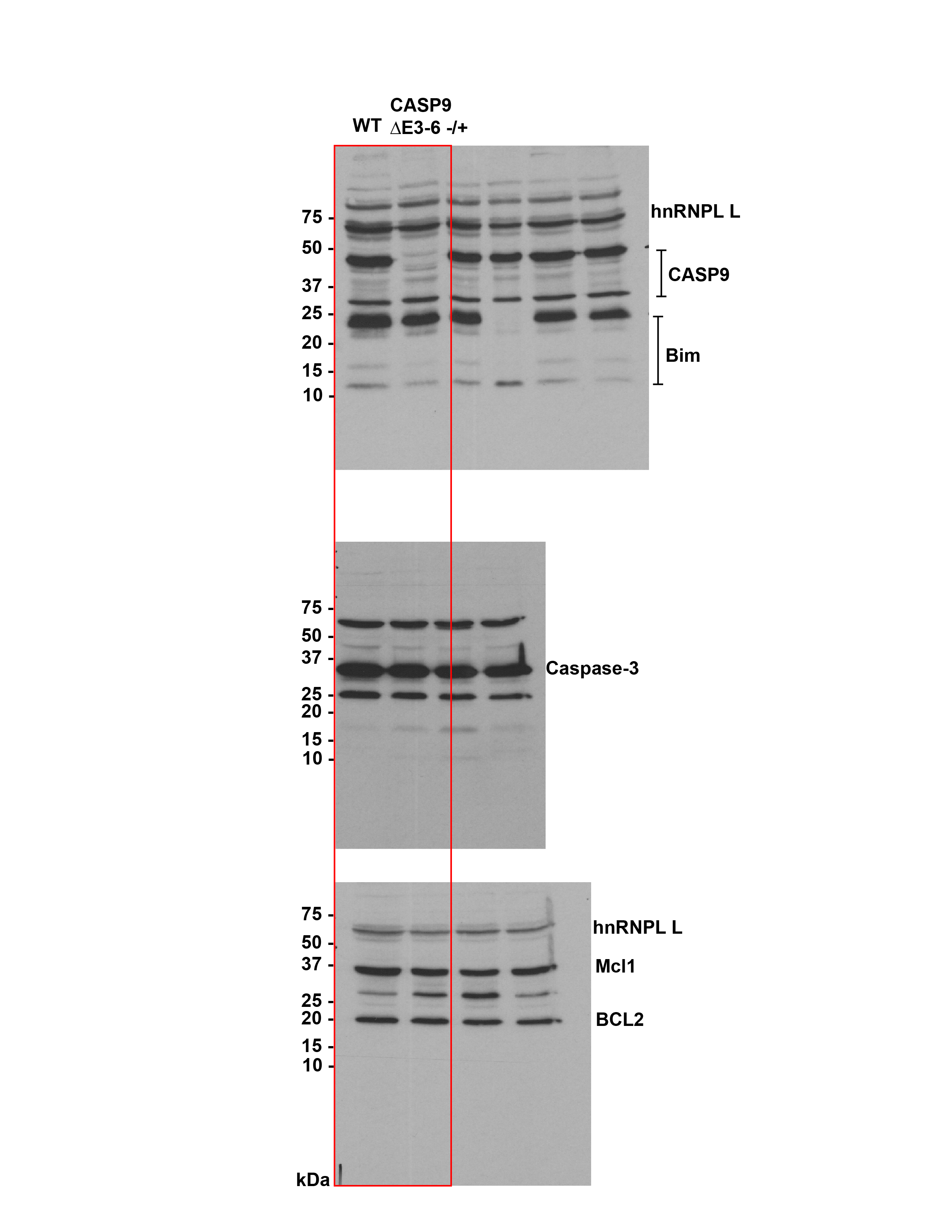

Supplement: Figure 5—figure supplement 1—source data 1. [file elife-80953-fig5-figsupp1-data1.zip › Figure 5 - figure supplemental 1 - source data 1 - CASP9 clone - labeled.jpg]

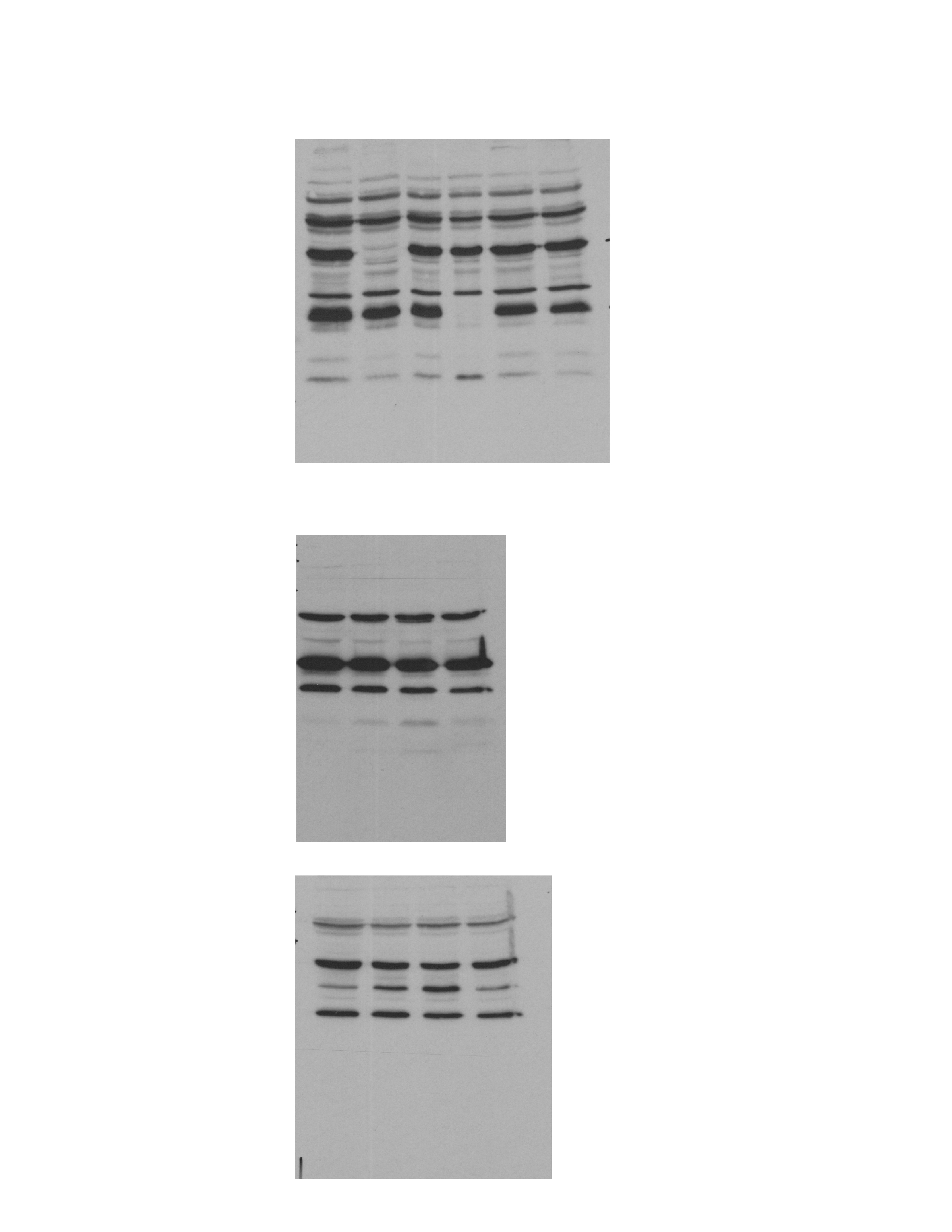

Supplement: Figure 5—figure supplement 1—source data 1. [file elife-80953-fig5-figsupp1-data1.zip › Figure 5 - figure supplemental 1 - source data 1 - CASP9 clone - Unlabeled.jpg]
